# Efficacy of the Arteriovenous Loop for Free Flap Reconstruction in Patients with Complex Limb Trauma: Case Series and Literature Review

**DOI:** 10.3390/medicina56110632

**Published:** 2020-11-23

**Authors:** Andrea Marchesini, Letizia Senesi, Francesco De Francesco, Pier Paolo Pangrazi, Andrea Campodonico, Rocco Politano, Michele Riccio

**Affiliations:** 1 Department of General and Specialties Surgery, SOD of Reconstructive Surgery and Hand Surgery, AOU “Ospedali Riuniti”, 60126 Ancona, Italy; andrea.marchesini@ospedaliriuniti.marche.it (A.M.); francesco.defrancesco@ospedaliriuniti.marche.it (F.D.F.); pierpaolo.pangrazi@ospedaliriuniti.marche.it (P.P.P.); andrea.campodonico@ospedaliriuniti.marche.it (A.C.); michele.riccio@ospedaliriuniti.marche.it (M.R.); 2 Department of Orthopaedics and Traumatology, Carlo Urbani Hospital, 60035 Jesi, Italy; rocco.politano@ospedaliriuniti.marche.it

**Keywords:** microsurgery, arteriovenous loop, complex limb trauma, free flap

## Abstract

*Background and objectives:* Complex limb traumas are commonly treated with microsurgical reconstruction and free flaps. However, complications are frequent in patients affected by a previous trauma or comorbidity, atheromasia and a single valid vessel. Free flap reconstruction is indeed a challenging procedure in complex injuries, which may increase the risk of limb ischemia. The Arteriovenous loop (AVL) technique may be considered an efficient alternative treatment. We herein report our procedure and previous research regarding the AVL method using a two-step reconstruction in cases of complex high-energy limb injuries. *Materials and Methods:* In this single center retrospective cohort study, all the patients from 2014 to 2018 who underwent to AVL reconstruction were assessed. A total of six patients were included in the study for traumatic limb trauma. The two-stage technique was performed each time. The age and sex of patient, the time between stage one and two, the length of AVL loop and rate of free flap success were evaluated. *Results:* A total of seven AVL reconstructions were performed. The mean age of patients was 36 years old. Eight free flaps were performed; six free flaps were transferred to the vascular loops. The average time between stage one and two was 13 days. The mean length of the pedicle was 25 cm for the upper limb and 33.7 cm for the lower limb. All the free flaps successfully take root. In one case, a surgical revision was required the second day post-operatory due to venous congestion. *Conclusions:* AVL is a useful and safe technique in microsurgical reconstruction which will prevent vascular complications. Our investigations suggest the efficacy and feasibility of a two-step intervention in acute post-traumatic events. A single-step procedure should be preferred in chronic situation and oncologic reconstruction.

## 1. Introduction

Complex limb traumas are characterized by pluri-structural lesions and are often caused by high-energy injuries. A damaged limb with poor vessels, represents a remarkable reconstructive challenge for microsurgeons. In these settings, crush injuries involve complex soft tissue as well as bone reconstruction with transfer of microvascular free tissue, particularly in lower limb traumas.

This type of injury is mostly caused by insufficient recipient vessels due to the high energy of trauma, arterial occlusive disease, previous accidents or surgery [1], as well as being attributable to the susceptibility of vessels in the defective area or to the dynamics of the injury. It is fundamental to operate away from this area to prevent risk of fibrosis, vessel fragility, and arterial spasms [2]. The high energy of trauma changes and affects all layers of the vessel wall, extending well beyond the site of the original injury [3].

A free flap transfer in a ‘mono-vessel’ patient is a detrimental and complex event due to the microsurgical technique (thrombosis, limb ischemia time) and blood flow depletion that may induce irreversible ischemic injury. A significant increase in thrombotic occurrences has been observed in the use of human vein grafts [4,5,6,7,8]. Therefore, the arteriovenous loop (AVL) technique may prove efficient in potential risk of thrombotic events identifying vessels that are distant from the trauma area using long vein grafts, which will be subsequently divided for tension-free anastomosis to free flap vessels.

AVL construction and free tissue transfer can be conducted via a single-step procedure, or staged intervals. The research has reported the application of these procedures with differing results, adverse outcomes and morbidity [9]. A single-step procedure yielded positive results not only from a physical and psychological perspective but also from an operative viewpoint, as it is possible to work through a clear field, stimulating bone union and stability diminishing scarring and healing time, consequently producing less infection and shorter hospital stay. However, a two-step intervention may be generally safer, in particular for serious-risk patients affected by complex injuries. Moreover, the two-step procedure offers a more reliable loop and a diminished failure risk due to its potential to distend the vessel at a physiologic pressure.

Threlfall et al. [10] presented the microsurgical vascular loop procedure in 1982 and Grenga et al. promoted it in 1987 [11], defining it as an efficient means for tissue transfer to recipient areas lacking in appropriate vessels for microsurgical anastomosis.

It is characterized by a temporary end-to-side vein graft between the artery and the vein (A/V fistula). The great saphenous vein or the cephalic vein harvested ipsi or contralaterally are typically used for vein grafts. The use of the lesser saphenous vein is also described herein [12,13].

**Figure 1:**
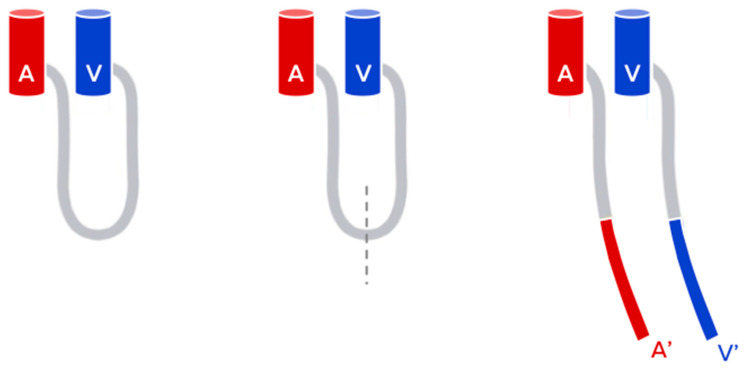
The Arteriovenous loop (AVL) technique.

If an adequate vascular flow within the loop is achieved, then it is possible to bisect the loop midway and then proceed to the anastomoses of the new vessels to the donor vessels in an end-to-end manner, using the arterial inflow and venous outflow for the free flap (Figure 1).

AVL enables anastomoses outside the injury area or may counterbalance the short pedicles which will consequently mitigate the tension and thrombosis hazard in the upper and lower extremities [10,11,14,15]. Besides promoting tension-free anastomoses, AVL will facilitate vascular distention at physiological pressures, thus assisting vein graft patency prior to tissue transfer as well as lowering the ischemia time of the vein graft [16].

This straightforward procedure will enhance leakage repair and remove twists in the vessels before transferring the free tissue, also enabling a rapid management of patency [1].

Vein grafts may present with numerous complications, hence the introduction of arteriovenous loops to diminish such events, especially in complicated cases of free flap transfer concerning the head and neck [17,18], trunk [19], upper limb [20,21,22], and the lower limb [23,24]. The loops create a low-resistant, high-flow, long arteriovenous shunt to the surrounding healthy vessels that may be instantly divided in a one-stage intervention or shortly after in a two-stage or delayed loop intervention, to offer optimal recipient vessels in proximity to the defective area [23].

Ample research has been conducted on the use of the arteriovenous loop in a one or two-stage procedure, but its indications have yet to be established.

The aim of our study is to conduct a clinical series regarding complex microvascular reconstructions of the extremities employing vascular loops as recipients. Additionally, we consider the indications and contraindications.

## 2. Materials and Methods

### 2.1. Patient Database

From 2014 to 2018, a total of seven consecutive patients underwent microsurgical AVL reconstruction. One patient (N#3) presented with a trophic ulcer by peripheral arterial occlusive disease (no limb trauma), and was hence excluded from the study. AVL procedures were performed for acute or delayed trauma cases due to absence or incompetent healthy recipient vessels in the surrounding trauma area. Six patients were affected by mangled limb injury, three patients were male and three were female.

In this specific cohort of patients, hyperbaric therapy was performed in all cases in order to promote the eradication of infection and the early demarcation of necrotic skin and tissues [25].

Preoperatory angiography or angio-Ct scan on injured limb was performed, to assess vessel status. Prior to the AVL procedure, preoperatory color doppler examination of proper vein graft was mandatory.

Absolute exclusion criteria for graft selection were:

- Vein size < 3 mm or > 10 mm;
- Sign of thrombosis or vein ectasia;
- Aftermaths of varices and phlebitis.

We focused on free flap interventions, considering the time between stage one and two in an AVL technique, length of AVL loop and rate of free flap success.

### 2.2. Surgical Procedure

All AVLs were produced by harvesting the contralateral great saphenous vein for the lower limb and the cephalic vein for the upper limb. We flushed the vein grafts with heparinized and Hartmann’s solution to ensure distension prior to anastomosis and presence of leakage. After graft yielding, an arteriovenous fistula was created, by a microsurgical end-to-side graft suture on large caliber vessels, away from the injured zone. Moreover, the central area of the arteriovenous loop was positioned in proximity to the lesion. Clamps were held until completion of arterial and venous anastomosis. No distal ischemic effects were noted. The vein loops were monitored after clamp release for uniform distension, crimps or twists. An adequate flow was ensured before inserting the AV loop into the limb. All cases were treated using a two-step technique. We monitored the AVL daily with an audible Doppler probe. Antiplatelet and anticoagulant prophylactic doses were systematically administered during the postoperative period of the loop construction. The second stage was initiated after approximately two weeks.

The AVL was dissected at its apex, thus creating two new vessels, connected to the vein and artery, respectively. Finally, we micro-surgically connected the free flaps end-to-end with the two vessels of the former loop. We initiated monitoring of the flap post-surgery every hour for the first 24 h and proceeded as such for the remainder of the patient’s stay, decreasing intensity after the first 24-h period. Monitoring involved physical inspection of flap warmth, turgor, capillary refill and color, as well as testing the vascular pedicle via Doppler examination.

We administered pharmacological treatment such as low molecular weight heparin (LMWH), unfractionated heparin (UFH), acetylsalicylic acid (ASA) and dextrans (DX) to lower the potential occurrences of thrombotic events and flap necrosis.

Specifically, the antiaggregant therapy was initiated after the first stage, with administration of ASA 100 mg once a day for thirty days. Prophylactic anticoagulant therapy was administered in the post-operatory setting after AVL preparation with subcutaneous LMWH (4000 UI once a day) until the second stage. After the flap settings in the second stage, UFH was started at 10.000 UI dilute in 50 cc saline solution in continuous intravenous infusion with syringe pomp at 2.1 cc/h, protracted for ten days and then replaced again by LMWH for fifteen days. DX was also started with IV UFH and protracted for 7 days (500 mL 2 cc/h in continuous intravenous infusion).

## 3. Results

The results are summarized in Table 1. A total of seven consecutive AVL interventions were performed from 2014 to 2018 in our hospital. The study included three males and three females with an average age of 36 years, presenting with traumatic limb injuries. We performed the reconstructions in a two-step procedure on two upper limbs and four lower limbs. Eight free flaps were performed, and six free flaps were transferred to the vascular loops. Four flaps were transferred according to the high-energy trauma of the upper limb and four flaps for the lower limb. The flaps transferred were the following: two Antero-lateral tight flaps (ALT), one latissimus dorsi muscle (LDM) free flap, four fibular free flaps (FF) and one gracilis free flap (GF).

The recipient arteries used in the AVL reconstruction were the brachial arteries in two patients, the popliteal artery in one patient and the femoral artery in three patients.

All AVLs were performed in a two-step intervention and the greater saphenous vein was employed in four patients and the cephalic vein in two patients.

**Figure 2:**
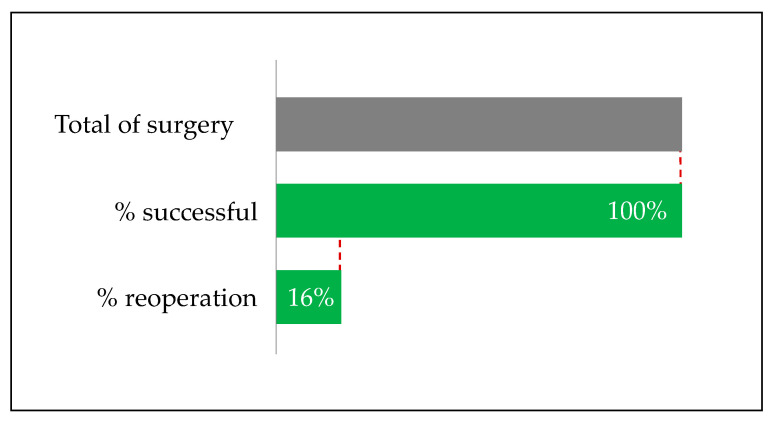
All the AVLs performed yielded successful outcomes. In one case, a surgical revision was required the second day post-surgery.

The time occurring between the one and the two-stage technique in AVL reconstruction was 13.6 days (according to the literature recommendations). We achieved completely positive outcomes (100%) in patients submitted to the two-stage procedure. In 16.6% (1/6) of the cases, a further surgical intervention was necessary following loop reconstruction and re-exploration of the anastomosis. Finally, all the free flaps successfully took root (no flap failure, no other surgical procedures required, no post-operative major complication) (Figure 2). Initially, in two patients (#1 and #4), free flap was performed to provide muscular and cutaneous reconstruction and coverage (ALT and GF + ALT respectively). Secondly, AVL reconstruction was intended for free FF in bone reconstruction. In the remaining cases, the AVL technique was performed directly for soft tissue reconstruction (#2 ALT FLAP, #6 LDM) or bone reconstruction (#5,7 FF). The average length of the AVL was 25 cm for the upper limb and 33.7 cm for the lower limb. In patient #2, revised AVL for thrombosis at day 2 post-operation was required.

We herein report details of two cases of the AVL technique, one in the upper limb and one in the lower limb.

### 3.1. Case Study #1

**Figure 3:**
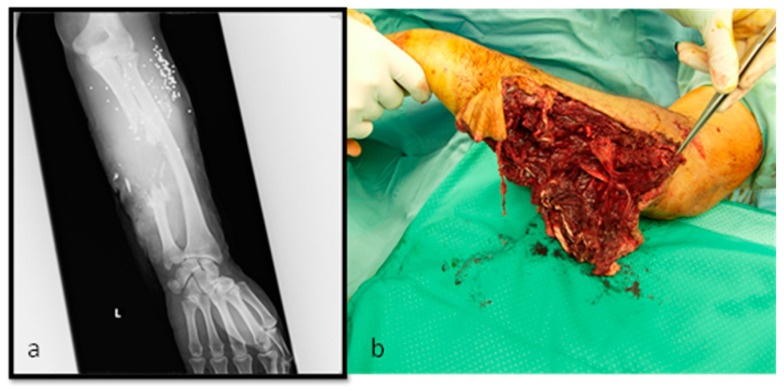
M.E. 50 years old, (**a**) initial x-ray and (**b**) clinical status, classified as IIIC G/A score.

M.E., 50 years old (y/o), ballistic trauma on upper left arm (Patient #4). The patients reported important soft tissue damage on extensor and flexor muscles on the ulnar side. The ulnar neurovascular bundle was destroyed (ulnar nerve and arterial gap 10 cm). X-rays displayed important ulnar bone loss (Figure 3a,b).

Initially, damage control was performed. An accurate debridement of injured and mangled muscular and tendon tissue is fundamental. Bone debridement and fixation were performed through external fixator. Therefore, Vacuum Assisted Therapy (VAC) was positioned, and broad-spectrum antibiotic therapy was initiated.

**Figure 4:**
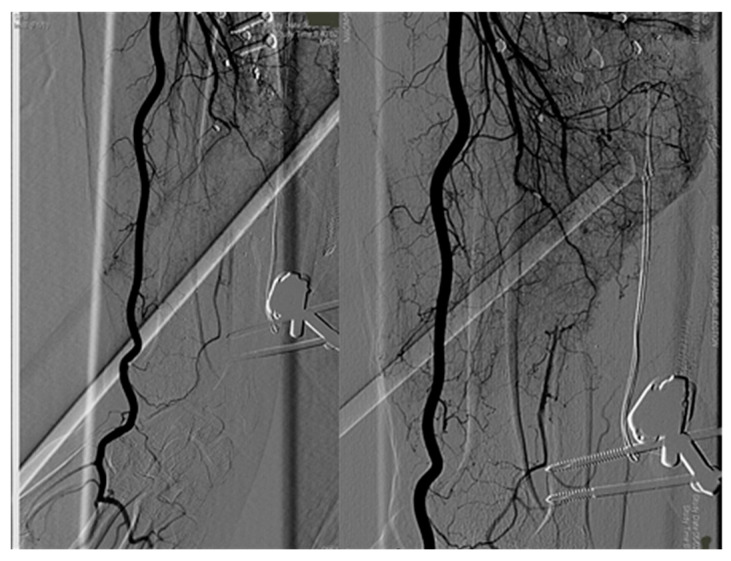
Angiography showed ulnar artery avulsion and interruption.

Post-operative angiography displayed only radial artery blood flow (Figure 4).

**Figure 5:**
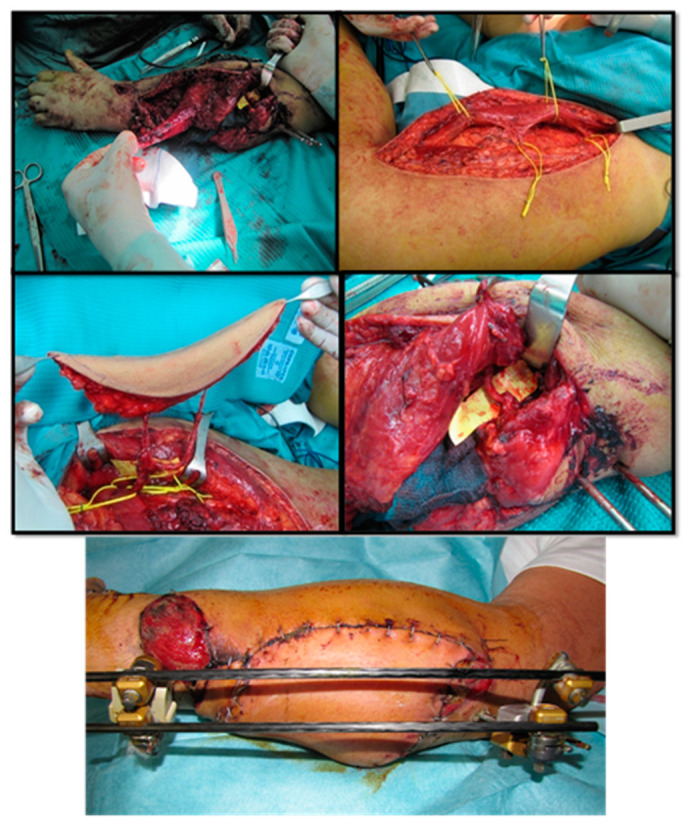
Initial soft tissue reconstruction. ALT free flap was anastomosed end-to-side with radial artery. Gracilis free flap was anastomosed end-to-and with ulnar artery interrupted proximally.

Firstly, we planned soft tissue coverage with gracilis and anterolateral tight free flaps. Additionally, we performed ulnar nerve reconstruction through the sural nerve graft, transposition of brachioradialis pro extensor pollicis lungus to restore extensor function (Figure 5).

**Figure 6:**
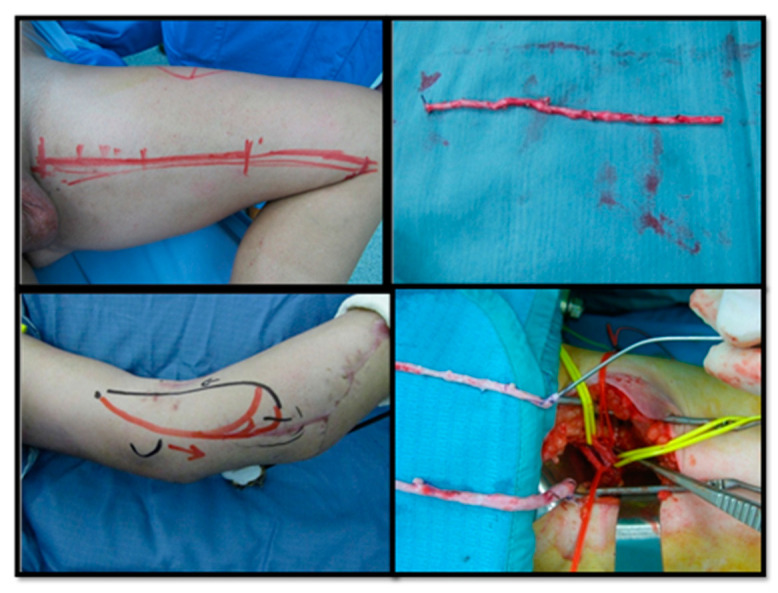
Harvested from great saphenous vein and anastomosed end-to-side with Brachial vein and Artery.

After one month, the reconstruction of the ulnar bone loss with a fibular free flap was planned. To avoid the steal phenomenon and ischemic upper limb damage, we performed AVL anastomosed on the Brachial bundle (Figure 6).

**Figure 7:**
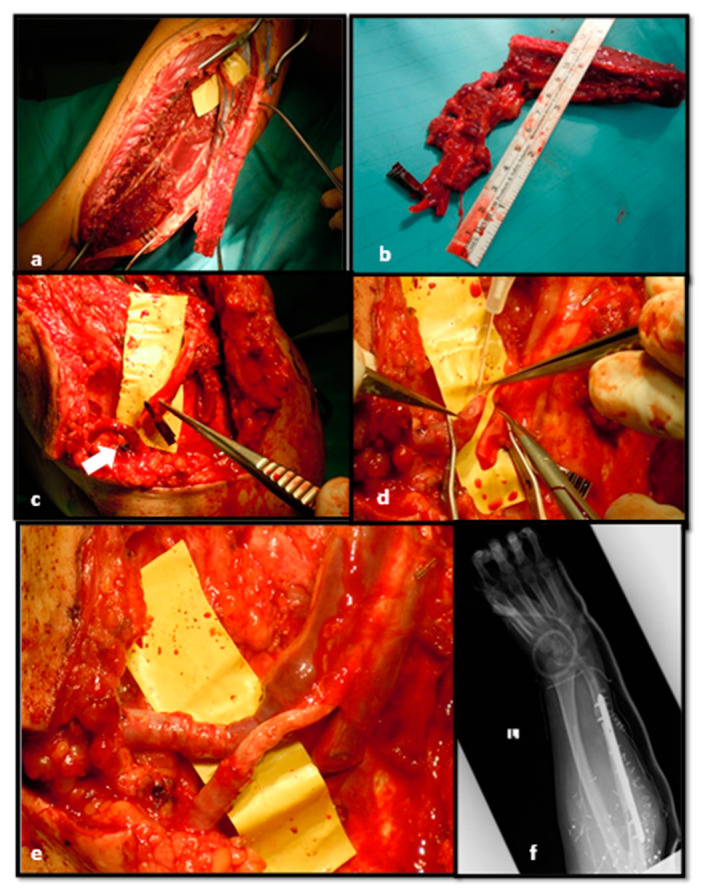
Free flap harvested from contralateral leg (**a**,**b**);Fibular pedicle placed near AVL (arrow) (**c**); AVL bisection at the centre (**d**); End-to-end fibular peduncle anastomoses on AVL (**e**); Post-operative x-rays after bone fixation (**f**).

After 14 days, we performed bone reconstruction by contralateral free fibular flap and anastomosed the vessels on AVL, centrally bisected in two parts (Figure 7).

**Figure 8:**
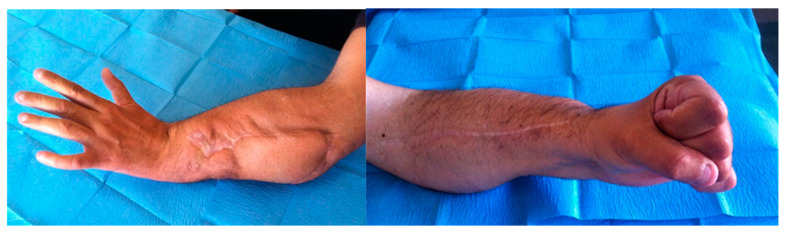
Patient #4, final outcome.

After surgery, our therapists commenced immediate post-operative physiotherapy with daily manual treatment focusing on active and passive movements to restore extensor and flexor function as well as prono-supination. A committed physiotherapic programme was continued on an outpatient basis. The final outcome, seven months after surgery (Figure 8), showed adequate restoration of hand and wrist functions.

### 3.2. Case Study #2

**Figure 9:**
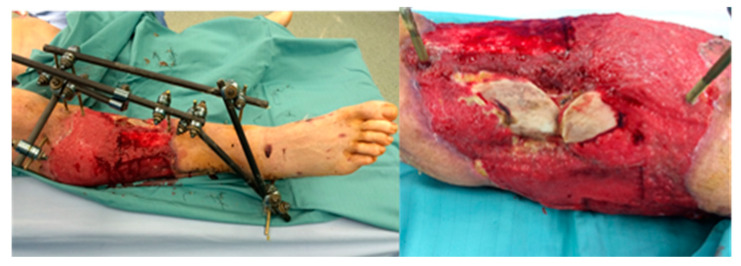
Post hyperbaric therapy viable tissue was observed but superficial tibial bone necrosis was defined.

M.L., 23 y/o. Motorcycle crush with open tibial fracture (3C Gustilo-Anderson Score) with soft tissue damage and bone exposure (Patient #6:). After damage control (external fixator location and soft tissue accurate debridement), patient underwent antibiotic and hyperbaric therapy. Subsequently, a partial tibial anterior bone necrosis was observed (Figure 9).

**Figure 10:**
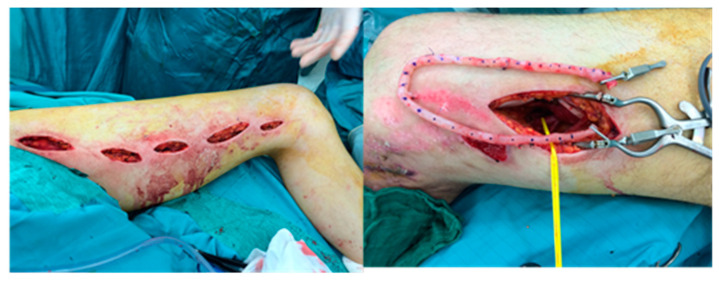
First stage of AVL technique, great saphenous vein harvested from contralateral legand attached end-to-side to femoral artery and vein.

The posterior tibial artery was compromised. To avoid ischemic damage to the lower limb, we planned soft tissue coverage with latissimus dorsi free flap using a two-stage AVL technique. Initially, we harvested the great saphenous vein from the contralateral leg. AVL was performed on the femoral vein and artery, 10 cm above the knee (Figure 10).

**Figure 11:**
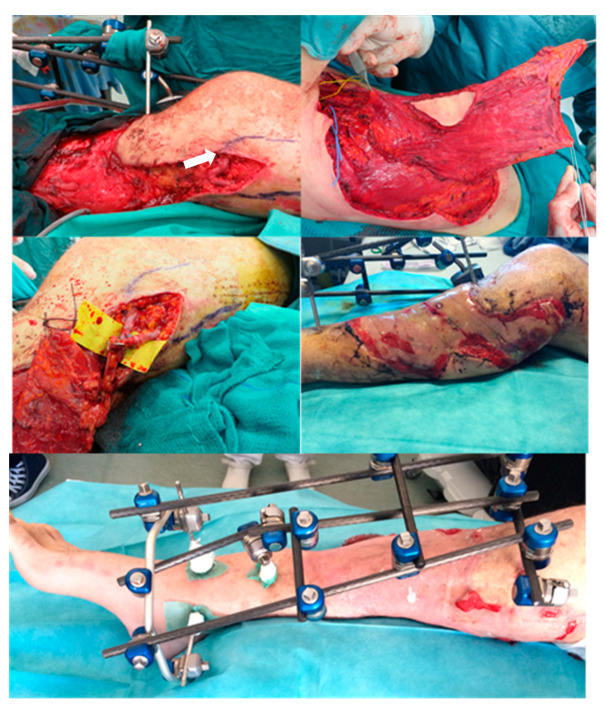
The midpart of the AVL (arrow) near the injured zone. Latissimus dorsi free flap was harvested and the pedicle anastomosed end-to-end with AVL. The muscle was covered with a dermal substitute. Final result three months later after skin graft positioning.

After 13 days, we performed a latissimus dorsi free flap with an end-to-end anastomosis on AVL, previously dissected centrally. The remnant site of the muscular flap was covered with a dermal substitute. The final outcome three months after skin graft is shown in Figure 11.

## 4. Discussion

Limb amputation determines important functional restrictions as well as significant social stigma. In complex limb injuries, soft tissue reconstruction with free flap preparation is frequently required, but positive flap outcomes are, however, strictly related to the presence of healthy recipient vessels. The surgeon is often obliged to resort to microsurgical reconstruction of a limb presenting inadequate recipient vessels. Moreover, healthy vessels are not to be located within close range of the defective area, especially regarding extensive traumatic injuries related to degenerative arterial disease or in patients with a history of past traumatic events.

This clinical context requires a sufficient length of vascular pedicles. However, using simple vein grafts alone may cause relevant complications. To solve this problem, the concept of the temporary arteriovenous fistula or vascular loop was developed [11]. AVL in selected patients surmounted the limitations of using vein grafts alone. The vascular loop is attached end-to-side to a large caliber proximal recipient vessel and stimulates the low-resistance and a high-flow system in proximity to the defective area. The division of the loop permits optimal inflow and outflow for the free flap and facilitates adequate accommodation of the corresponding length of the artery and the venous ends of the loop. According to Cavadas [26], the single-step procedure presents more disadvantages from a logistic and economic perspective compared to the two-step free flap procedure. This is indicated in patients with cardiopulmonary problems (arteriovenous fistulas typically lower arterial pressure but will raise peripheral and central venous pressure and increase the ventricular preload). Cho et al. [1] also advocate the use of a one-step procedure. Moreover, in complex limb injuries, further delaying flap transfer to the loop to several days may ensure safer practice. In case of AVL obstruction, the flap remains retrievable and a second loop may be reconstructed. We propose the two-stage vascular loop technique on account of the benefits obtained post-vein-arterializations, also considering a reduced occurrence of adverse events related to “serial” anastomoses. These advantages allowed for a clear vision of potential complications in the loop prior to flap transfer. Moreover, Silva GB and colleagues [27] reported no statistical difference between the single-stage and two-stage procedure.

We reported a case of AVL thrombosis requiring revision two days after surgical intervention. In contrast to Tremp et al. [28] in our series we observed no free flap failure after a two-stage reconstruction. However, delayed two-stage arteriovenous loops may yield unfavorable adverse outcomes, especially in patients affected by cardiac failure with low stroke volume as a consequence of the increased preload, which may provoke serious heart failure or a myocardial infarction as well as potentially inciting the steal phenomenon. According to our series, a recent metanalysis reported that upper and lower free flaps using the two-step ALV method were most commonly used in mangled limb injuries [11]. Furthermore, a two-step procedure including shorter and simpler interventions seems to favor patients with significant comorbidities (noncardiac). A patient with a past history of multiple operations will show fatigue and reluctance regarding flap loss. In agreement with Cavadas, we suggest a staged procedure in complex cases to ensure more safety [20]. Lind et al. [29] also proposed a more accessible two-step technique enabling monitoring of the fistula for several days to certify sufficient blood flow for the free flap [10]. Henn et al [30], in a recent paper, reviewed a unique cohort (the largest published in the literature) of 101 AVL interventions. A two-stage arteriovenous loop reconstruction lowers the risk of postoperative complications compared to a single-stage arteriovenous loop reconstruction and is proposed specifically in complicated cases due to shorter operative times. Nevertheless, it should be noted that the second stage is to be conducted within 10 to 14 days (average time in our cohort was 13 days), to prevent difficulties in dissecting the loop compromised by the presence of scar tissue. The increased risk of thrombosis and shorter operative times does not allow for reendothelialization to occur in the anastomotic lines and in the graft itself. In our opinion, the one-stage technique is recommended in an oncologic or non-traumatic context and in cardiopathic patients. Filling the graft with heparin before initiation of the anastomoses is imperative to prevent twisting of the long vein graft. Furthermore, careful preparation of the tissue bed is fundamental; the loop will rest therein during the two-stage reconstruction to avoid kinking or compression of the loop in the subcutaneous tissue and prevent thrombosis.

Our study has certain limitations, such as a small sample size, a lack of homogeneity and its retrospective nature. Thus, our results can be considered preliminary. More observational and large-scale studies should be conducted to confirm these preliminary results.

## 5. Conclusions

The AVL procedure generates a neovascular system and is efficient in cases of unforeseen vascular complications within an inappropriate recipient area, especially relevant for complex limb traumas. Our studies advocate an arteriovenous loop as an innovative tool to address the complications that may arise from micro-reconstructions in poorly vascularized lower extremities.

Indications for a single-stage or two-stage reconstruction need to be clearly determined, and in light of our findings, along with the existing literature, we affirm positive outcomes and preference for a reconstruction performed in two separate stages in complex limb traumas.

## Figures and Tables

**Table 1:** Demographic characteristics of patients enrolled are shown.

Patient	Sex	Age	Type of Trauma	Site of Trauma	Free Flap	Loop Length (cm)	Recipiental Artery	Time for 2 Stage (days)	Side Effects
#1	F	22	Crush-Burn Injury (mechanical press)	Right hand	(1) ALT (2) “loop” FF	25	Brachial artery	13	None
#2	F	40	Derotative osteotomy in post-traumatic chronic tibial non-union	Right leg	“loop” ALT	30	Popliteal Artery	21	2nd day loop revision (thrombosis)
#4	M	51	Balistic Trauma	Left arm	(1) ALT+GF (2) “loop” FF	25	Brachial Artery	12	None
#5	M	37	Open tibial fracture (3C GA) motorcycle accident	Right leg	“loop” FF	30	Femoral Artery	11	None
#6	M	23	Open tibial fracture (3C GA) motorcycle accident	Right leg	“loop” LDM	35	Femoral Artery	13	None
#7	F	44	Throw herself from high	Left leg	“loop” FF	40	Femoral Artery	12	None

Patient #3 was excluded from the study (non- traumatic limb). Patients #1 and #4 initially underwent free flap intervention for muscolo-cutaneous reconstruction, and secondly the AVL permitted bone reconstruction. Abbreviations: ALT: Antero-lateral tight flap; FF: fibular flap; LDM: latissimus dorsi flap; GF: gracilis flap.
